# Evaluating plasma renin activity as a monitoring parameter for mineralocorticoid treatment in dogs with primary hypoadrenocorticism

**DOI:** 10.1186/s12917-026-05645-y

**Published:** 2026-08-08

**Authors:** Soraya Arrigoni, Livia A. Harburger, Elisabeth Neubert, Barbara Riond, Helen Evans, Stefan Unterer, Felicitas S. Boretti, Nadja Sieber-Ruckstuhl

**Affiliations:** 1 Clinic for Small Animal Internal Medicine, Vetsuisse Faculty, University of Zurich, Zurich, Switzerland; 2 Clinical Laboratory, Vetsuisse Faculty, University of Zurich, Zurich, Switzerland; 3 Nationwide Specialist Laboratories, Cambridge, UK; 4 Bessy’s Kleintierklinik, ZN IVC Evidensia Schweiz AG, Watt/Regensdorf, Switzerland

**Keywords:** Primary hypoadrenocorticism, Plasma renin activity, Desoxycorticosterone pivalate treatment, Canine

## Abstract

**Background:**

In human medicine, plasma renin activity (PRA) is used to assess mineralocorticoid treatment adequacy in hypoadrenocorticism, with elevated levels indicating under-replacement and suppressed levels over-replacement. In dogs with primary hypoadrenocorticism, PRA is increased before treatment and decreases with treatment, but its value as a monitoring parameter remains uncertain. The objective of this study was to evaluate PRA as a marker to guide mineralocorticoid dosing in dogs with primary hypoadrenocorticism. A total of 52 PRA measurements were collected from 28 dogs during scheduled desoxycorticosterone pivalate (DOCP) treatment visits. All dogs had received DOCP and prednisolone for at least five months. PRA was measured by ELISA detecting angiotensin I.

**Results:**

Median PRA was 1.6 ng/mL/h (range, 0–19.2), with 19 values above, 17 within, and 16 below the reference interval. PRA showed moderate correlations with potassium (*p* = 0.009, 𝛒 = 0.36), sodium (*p* = 0.02, 𝛒 = -0.33), the sodium-to-potassium (Na/K) ratio (*p* = 0.006, 𝛒 = -0.38), and DOCP dose (*p* = 0.02, 𝛒 = -0.34), while urine specific gravity correlated with DOCP dose (*p* = 0.01, 𝛒 = -0.50). Compared with visits with decreased PRA, visits with increased PRA had a significantly lower Na/K ratio (*p* = 0.048). Increased PRA occurred more frequently during visits with concurrent disease (*p* = 0.005); concurrent disease was also associated with higher creatinine concentrations (*p* = 0.023) and lower DOCP doses (*p* = 0.004). In subgroup analyses, chronic enteropathy was associated with higher potassium concentrations (*p* = 0.003), lower Na/K ratios (*p* = 0.009), and higher DOCP doses (*p* < 0.001), whereas chronic kidney disease was associated with higher creatinine concentrations (*p* < 0.001) and lower DOCP doses (*p* < 0.001). Clinical assessment and PRA status were concordant in 48% of visits.

**Conclusions:**

PRA may provide additional information for monitoring mineralocorticoid treatment in dogs with hypoadrenocorticism treated with DOCP. However, its interpretation can be challenging, as it often does not match electrolyte results and both PRA and electrolytes may be affected by other diseases. Therefore, PRA should be interpreted as a supportive parameter within the clinical context.

**Supplementary Information:**

The online version contains supplementary material available at https://doi.org/10.1186/s12917-026-05645-y.

## Introduction

Primary hypoadrenocorticism is an uncommon but clinically important canine endocrinopathy most commonly caused by immune-mediated destruction of the adrenal cortex, resulting in deficient glucocorticoid and mineralocorticoid secretion. The majority of dogs is presented with the typical electrolyte pattern of hyponatremia and hyperkalemia and require lifelong hormone replacement, with a combination of glucocorticoids and mineralocorticoids [1–3].

The pathophysiology of hypoadrenocorticism directly affects systems essential for maintaining vascular tone, fluid balance, and electrolyte regulation. One such system is the renin-angiotensin-aldosterone system (RAAS), a hormonal cascade critical for extracellular volume, potassium excretion, and blood pressure regulation [1, 3]. In healthy animals, renin, released by the juxtaglomerular cells of the kidney, catalyzes the conversion of angiotensinogen to angiotensin I, which is subsequently converted to angiotensin II by angiotensin-converting enzyme. Angiotensin II stimulates aldosterone release from the adrenal cortex, completing a negative feedback loop that suppresses further renin secretion. In primary hypoadrenocorticism, the absence or insufficiency of aldosterone eliminates this feedback mechanism, resulting in persistently elevated plasma renin activity (PRA) as a compensatory response to hypotension, hypovolemia, and electrolyte imbalances [4–6]. In human medicine, PRA is often used to assess the adequacy of mineralocorticoid replacement. The theory is that elevated PRA reflects under-replacement, whereas suppressed PRA suggests over-replacement [7, 8]. However, the evidence is mixed, and clinicians frequently consider sodium, potassium and blood pressure together with PRA, when making dosing decisions, rather than relying on PRA alone. This approach enables individualized, physiology-based management and has been associated with improved clinical outcomes and reduced cardiovascular risk [8].

In veterinary medicine, dogs with untreated primary hypoadrenocorticism generally show increased PRA, which becomes suppressed following mineralocorticoid treatment [4, 5]. DOCP, a long-acting injectable mineralocorticoid, tends to produce stronger PRA suppression than fludrocortisone, a mineralocorticoid that has to be administered orally on a daily basis [5]. Despite these observations, it remains unclear how useful PRA is for routine monitoring and dose titration in dogs with primary hypoadrenocorticism. Over-replacement with DOCP is undesirable because excessive mineralocorticoid activity promotes sodium retention and potassium loss, which can lead to hypernatremia, hypokalemia, volume expansion, edema, and even cardiac enlargement or hypertension, especially in dogs with cardiac or renal comorbidities.

In daily clinical practice, DOCP dose adjustments are commonly guided by serum potassium and sodium concentrations, with elevated potassium levels prompting dose increases and low levels of potassium prompting dose reductions [9]. This pragmatic approach is widely used but may not fully reflect the dynamics of the RAAS or individual variability in treatment response.

The primary objective of this study was to evaluate the association between PRA and the adequacy of mineralocorticoid therapy, as assessed by electrolyte concentrations and clinical evaluation. Secondary objectives were to investigate correlations between PRA and electrolyte concentrations, associations with other treatment variables (including DOCP dose) and clinical assessment, as well as the impact of concurrent diseases.

We hypothesized that PRA would be suppressed in adequately or excessively treated dogs and elevated in those with suboptimal mineralocorticoid replacement.

## Materials and methods

### Study population

A prospective observational study was conducted and dogs with primary hypoadrenocorticism with electrolyte imbalances were included. Dogs were presented to the Clinic for Small Animal Internal Medicine, Vetsuisse Faculty, University of Zurich, between February 2022 and February 2024 for regular re-checks. The diagnosis of primary hypoadrenocorticism had been confirmed in all cases based on baseline and post-ACTH stimulation cortisol concentrations consistent with adrenal insufficiency (< 2 µg/dL), in conjunction with compatible clinical signs, hyponatremia and/or hyperkalemia, and, when available, elevated plasma ACTH concentrations. In all dogs, prior administration exogenous glucocorticoids or progestogens had been excluded, as defined in the ESVE ALIVE guidelines [10]. Eligible dogs had been treated for at least five months prior to enrollment and were on a stable treatment regimen consisting of DOCP and prednisolone. Dogs newly diagnosed with primary hypoadrenocorticism or those receiving fludrocortisone were excluded.

At the time of diagnosis of primary hypoadrenocorticism all dogs were started on DOCP and prednisolone according to a standardized treatment protocol at our institution. The initial DOCP dose was 1.5 mg/kg administered subcutaneously every 28 days. Based on serum electrolyte concentrations measured on days 14 and 28 after the first injection, the DOCP dose was adjusted. If hypokalemia was present on day 28, the next injection was delayed by at least one week, until potassium levels were within the reference interval. For each week of delay, the DOCP dose was reduced by 10%. In cases of hyperkalemia on day 28, the next dose was administered immediately and increased by 5–10%. If potassium concentrations were within the reference interval on day 28, the same DOCP dose was repeated. Sodium concentrations were also monitored at each recheck; however, DOCP dose adjustments were primarily guided by potassium levels unless marked hyponatremia was observed. After the second DOCP injection, dogs were typically re-evaluated on the day of the subsequent injection (day 28), and the dose was again adjusted as described. Following the third injection, blood recheck intervals were extended to every 3–4 months, with DOCP injections administered every 28 days in between without interim blood monitoring.

The initial prednisolone dose administered in the hospital at the time of diagnosis ranged from 0.5 to 1 mg/kg IV every 6 to 12 h, depending on the severity of clinical signs, and was continued for 12 to 48 h. At discharge, the dose was reduced to 0.5 mg/kg/day orally and further decreased to 0.25 mg/kg/day after three days, continuing until the 14-day recheck. At that point, the dose was gradually tapered, typically by 50% every seven days, until a maintenance dose of approximately 0.05 to max. 0.1 mg/kg/day was reached.

### Clinical monitoring and sampling protocol

Each dog was evaluated during a scheduled recheck visit for its routine DOCP injection (± 1 day). Owners were instructed to bring a urine sample to the hospital, and the urine specific gravity (USG) was measured. At each visit, a comprehensive clinical history was obtained, and the current doses of DOCP and glucocorticoids were recorded. The presence of concurrent diseases was recorded, and any chronic complaints potentially arising from these conditions were noted. Only clinically stable animals were included in the study; animals with acute or newly emerging clinical signs potentially related to a concurrent disease were excluded. Additionally, any adjunct therapies required due to the concurrent diseases were documented. Non-invasive blood pressure measurement (Dinamap Carescape V100 Vital Signs Monitor) was performed after an appropriate acclimatization period. Blood pressure measurements were conducted according to the ACVIM consensus guidelines, and only dogs with at least five reliable readings were included [11]. A systolic blood pressure > 160 mmHg was defined as hypertensive [11]. Subsequently, a physical examination was performed. Venous blood samples were collected prior to DOCP administration via jugular or cephalic venipuncture into both EDTA and serum tubes. Serum was separated within 30 min and immediately analyzed for sodium, potassium, and creatinine concentrations using an ion-selective electrode analyzer in the in-house laboratory. Electrolyte values were classified as hypo-, normo-, or hyperkalemic and hypo-, normo-, or hypernatremic according to institutional reference intervals (potassium: 4.3–5.3 mmol/L; sodium: 145–152 mmol/L). For measurement of PRA, EDTA blood was collected into pre-chilled tubes, immediately placed on ice, and centrifuged at 4 °C. The resulting plasma was aliquoted and stored at − 80 °C until batch shipment on dry ice for analysis. PRA was measured in two batches: the first 18 months after study initiation and the second 18 months later. Consequently, sample storage time for PRA analysis did not exceed 18 months. Based on PRA results, the dogs were classified into three categories: normal, increased, or decreased PRA.

At each re-evaluation, every dog was assigned to a clinical impression category: good (appropriate mineralocorticoid dose), under-replaced (suspected mineralocorticoid underdosing), or over-replaced (suspected mineralocorticoid overdosing). This assessment was performed by one of two endocrinology specialists (NSR or FSB) prior to the availability of PRA results.

The clinical assessment was based on history (e.g., appetite, polyuria, polydipsia, vomiting, diarrhea), clinical findings (e.g., changes in body weight), and electrolyte concentrations (sodium and potassium), and was evaluated as independently as possible from glucocorticoid supplementation. Based on this evaluation, a recommendation was made to maintain, increase, or decrease the DOCP dose, which was then incorporated into the overall category assessment.

### Plasma renin activity measurement

PRA was measured at Nationwide Specialist Laboratories (Cambridge, United Kingdom) using a commercially available enzyme-linked immunosorbent assay (ELISA) that indirectly quantifies PRA through the rate of angiotensin I generation. The assay was performed according to the manufacturer’s instructions. The reference interval for PRA was defined as 0.22–2.4 ng/mL/h, based on historic validation and published canine reference data [12].

### Ethical considerations

All procedures were approved by the Cantonal Veterinary Office of Zurich (permission number, ZH203/2022) and conducted in accordance with guidelines established by the Animal Welfare Act of Switzerland.

### Statistical analysis

All statistical analyses were performed in R (v4.5.0; [13]) and GraphPad Prism (v10.4; GraphPad Software, San Diego, CA, USA). Normality was assessed using the D’Agostino–Pearson omnibus normality test. Because plasma renin activity (PRA) was non-normally distributed, associations between PRA and continuous variables were evaluated using Spearman’s rank correlation. Correlation strength was interpreted according to Cohen’s conventions (*r* < 0.10 very small; 0.10 ≤ *r* < 0.30 small; 0.30 ≤ *r* < 0.50 moderate; *r* ≥ 0.50 large) [14].

Due to repeated measurements from the same dogs across multiple timepoints, outcomes were analyzed using mixed-effects models with dog ID as a random intercept. Model choice was guided by outcome distribution and diagnostic assessment (e.g., residual normality, heteroscedasticity, and influential observations).

PRA was analyzed using Tweedie mixed-effects models with a log link fitted in glmmTMB to account for the right-skewed distribution [15]. Separate models were fitted with concurrent disease and clinical impression as fixed effects. Estimated marginal means and post-hoc pairwise contrasts were obtained using emmeans [16]. For Tweedie models, estimated marginal means were computed on the log scale and back-transformed for presentation; confidence intervals and hypothesis tests were performed on the log scale, and pairwise comparisons were reported as ratios with 95% confidence intervals and Tukey adjusted p-values. In addition to analyzing PRA as a continuous outcome, PRA values were categorized as decreased (below reference interval), normal (within reference interval), or increased (above reference interval), and the association of PRA category with other outcomes was assessed using mixed-effects models with PRA category as a fixed effect. Outcomes for which Gaussian assumptions were considered adequately satisfied (e.g., Na, K, Na/K) were analyzed using linear mixed-effects models fitted with lmer (lme4; [17]). Post-hoc comparisons were performed using contrasts of estimated marginal means. Results are reported as model-based effect estimates (beta, β) with 95% confidence intervals and Tukey adjusted p-values. Quantitative data are also summarized descriptively as median (range) in the tables where model results were reported. Concordance between PRA category and clinical impression was assessed descriptively (percent agreement). Statistical significance was set at *p* < 0.05.

## Results

### Animals

Twenty-eight dogs were enrolled in the study, including 10 males (10 castrated) and 18 females (15 spayed). The age at the time of inclusion in the study ranged from 2.5 to 14.5 years (median, 7.5 years), and body weight ranged from 3 to 50.2 kg (median, 12 kg). The following breeds were represented (numbers in parentheses indicate number of dogs): Mix-breed dog (7), Labradoodle (3), Jack Russell Terrier (3), Havanese (2), Australian Terrier (1), Berger Blac Suisse (1), Border Collie (1), Cane Corso Italiano (1), Chesapeake Bay Retriever (1), Clumber Spaniel (1), Dachshund (1), English Springer Spaniel (1), German Shepherd Dog (1), Golden Retriever (1), Labrador Retriever (1), Poodle (1), Spitz (1). Fourteen dogs were included once, whereas 14 dogs contributed multiple follow-up evaluations, with a median of 2 recheck visits per dog (range: 2–5), resulting in a total of 52 recheck visits available for analysis.

### Plasma renin activity

**Fig. 1:**
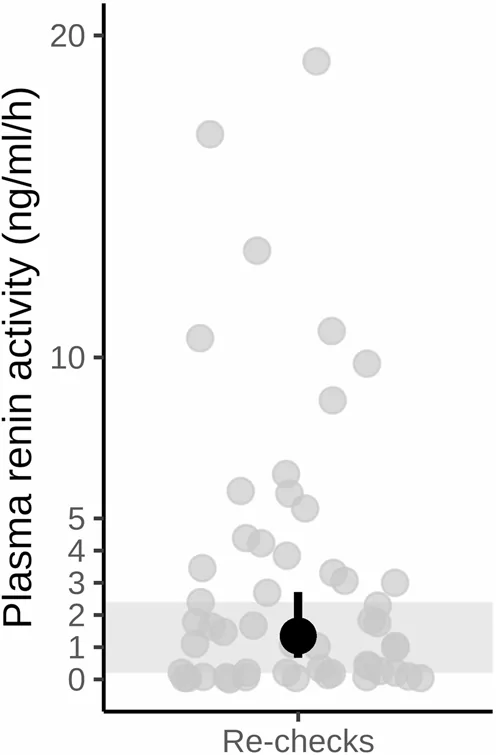
Scatterplots of plasma renin activity (PRA) of the 52 visits. Individual visits are shown as semi-transparent gray dots. Black circles represent estimated marginal means with vertical lines indicating 95% confidence intervals. The shaded gray area marks the PRA reference interval

A total of 52 PRA measurements were obtained on the day of scheduled DOCP administration (52 visits). The median PRA was 1.6 ng/mL/h (range: 0–19.2 ng/mL/h). According to the laboratory reference interval (0.22–2.4 ng/mL/h), 19 samples (36.5%) were elevated, 17 (32.7%) were within the reference interval, and 16 (30.8%) were below the reference interval (Fig. 1).

### Correlations

**Fig. 2:**
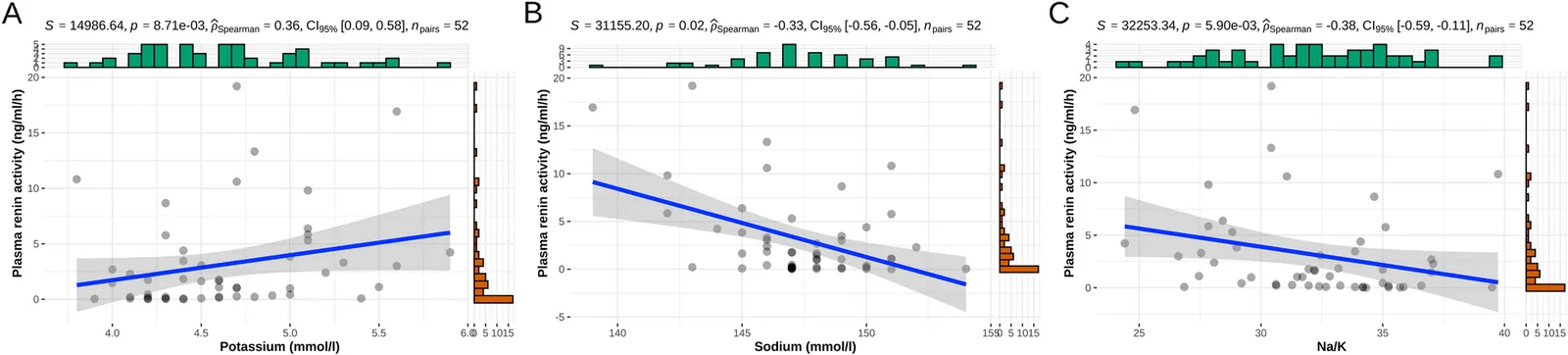
Spearman non-parametric correlation analysis between plasma renin activity (PRA) and electrolyte parameters. Scatterplots display the relationship of PRA with (**A**) potassium concentration, (**B**) sodium concentration, and (**C**) sodium-to-potassium (Na/K) ratio. Each plot includes individual data points (gray dots = visits), the Spearman correlation trend line (blue) with 95% confidence interval (shaded area), and marginal histograms of each variable. Plot titles display p-values (p), Spearman's correlation coefficient ($\:{\widehat{\rho\:}}_{\mathrm{Spearman}}$) with its 95% confidence intervals (CI95%) as a measure of the effect size and number of data pairs ($\:{n}_{\mathrm{pairs}}$). Significant moderate correlations are observed for potassium (positive correlation), sodium (negative correlation), and Na/K ratio (negative correlation). Statistical significance was defined as p < 0.05.

All performed correlations, including the *p*-value, Spearman’s 𝛒, 95% CI and the strength of the correlation, are presented in Table 1. Statistically significant, moderate correlations were observed between PRA and serum potassium concentration, PRA and serum sodium concentration, PRA and the sodium-to-potassium ratio, which are presented in Fig. 2. In addition, significant, moderate correlations were also observed between PRA and DOCP dose, as well as between DOCP dose and USG.

**Table 1:** Spearman correlations of PRA, electrolytes, and treatment parameters

Spearman correlation	*p*-value	Spearman’s 𝛒 (95% CI)	Strength of significant correlation	Number of pairs
PRA and potassium concentration	**0.009**	0.36 (0.09, 0.58)	Moderate	52
PRA and sodium concentration	**0.02**	-0.33 (-0.56, -0.05)	Moderate	52
PRA and Na/K ratio	**0.006**	-0.38 (-0.59, -0.11)	Moderate	52
PRA and DOCP dose	**0.02**	-0.34 (-0.56, -0.06)	Moderate	52
PRA and prednisolone dose	0.59	0.08 (-0.21, 0.35)	No correlation	52
PRA and USG	0.86	0.04 (-0.37, 0.44)	No correlation	25
PRA and systolic blood pressure	0.67	-0.08 (-0.41, 0.28)	No correlation	34
DOCP dose and USG	**0.01**	-0.50 (-0.75, -0.11)	Moderate	25
DOCP dose and systolic blood pressure	0.63	-0.09 (-0.42, 0.27)	No correlation	34
Prednisolone dose and USG	0.97	0.008 (-0.4, 0.41)	No correlation	25

*PRA* plasma renin activity, *Na/K ratio* sodium-to-potassium ratio, *USG* urine specific gravity, *DOCP* desoxycorticosterone pivalate, *CI* confidence interval

Correlation strength was interpreted according to Cohen’s conventions: *r* < 0.10 very small, 0.10 ≤ *r* < 0.30 small, 0.30 ≤ *r* < 0.50 moderate, and *r* ≥ 0.50 large [14]

Statistical significance was defined as *p* < 0.05

### Clinical examination, blood pressure measurements and urine specific gravity

At the 52 recheck visits, all dogs were clinically stable. The following mild clinical signs were documented (numbers in parentheses indicate the number of visits): mild polydipsia and polyuria (4), polyphagia (3), intermittent vomiting and diarrhea (4), soft stool (1), weight gain (2), intermittent reduced appetite (2), intermittent muscle stiffness (2), lip smacking (2), and presence of dandruff (1).

The study protocol included blood pressure measurement prior to invasive procedures and assessment of USG using owner-collected samples. However, blood pressure measurement was time-consuming and technically challenging due to dogs’ activity levels and scheduling constraints, resulting in incomplete data (32 of 52 visits). Of these, 12.5% were classified as hypertensive, and no significant differences in systolic blood pressure were observed between PRA groups (Table 2).

**Table 2:** Comparison of clinical and biochemical variables among PRA categories in dogs on mineralocorticoid therapy

	**PRA decreased**	**PRA normal**	**PRA increased**	**Comparison between groups** **Beta** ^**1**^ **(95% CI), p-value**
**Potassium (mmol/L)**				
Median (range)	4.3 (3.9-5.4)	4.6 (4.0-5.5)	4.8 (3.8-5.9)	Normal – decreased: 0.19 (-0.18, 0.55), 0.443
Mean (95% CI)	4.46 (4.23, 4.7)	4.65 (4.43, 4.88)	4.83 (4.61, 5.06)	Increased – decreased: 0.37 (0.00, 0.74), 0.052
Number of dogs	16	17	19	Increased – normal: 0.18 (-0.12, 0.49), 0.307
**Sodium (mmol/L)**				
Median (range)	148 (143-154)	148 (145-152)	146 (139-151)	Normal – decreased: -0.05 (-2.3, 2.2), 0.998
Mean (95% CI)	148 (146, 149)	148 (146, 149)	146 (145, 147)	Increased – decreased: -1.8 (-4.1, 0.48), 0.146
Number of dogs	16	17	9	Increased – normal: -1.8 (-3.9, 0.31), 0.108
**Na/K ratio**				
Median (range)	34 (27-39)	32 (27-37)	30 (24-40)	Normal – decreased: -1.5 (-4.2, 1.3), 0.403
Mean (95% CI)	33.4 (31.7, 35.2)	32 (30.3, 33.6)	30.6 (28.9, 32.3)	Increased – decreased: -2.8* (-5.6, -0.02), **0.048**
Number of dogs	16	17	19	Increased – normal: -1.3 (-3.7, 0.99), 0.351
**Creatinine (** **μmol/L)**				
Median (range)	81 (54-130)	76 (51-130)	96 (61-167)	Normal – decreased: 3.9 (-15, 23), 0.875
Mean (95% CI)	84.3 (71.2, 97.4)	88.2 (75, 101.4)	97.8 (84.5, 111.0)	Increased – decreased: 13 (-6.2, 33), 0.230
Number of dogs	5	15	17	Increased – normal: 9.5 (-5.0, 24), 0.248
**Urine specific gravity**				
Median (range)	1.020 (1.005-1.050)	1.031 (1.014-1.050)	1.035 (1.006-1.055)	Normal – decreased: 1.3 (-22, 25), 0.989
Mean (95% CI)	1027 (1015, 1040)	1029 (1013, 1044)	1035 (1023, 1048)	Increased – decreased: 7.8 (-13, 29), 0.620
Number of dogs	8	6	11	Increased – normal: 6.5 (-16, 29), 0.753
**Systolic blood pressure (mmHg)**				
Median (range)	136 (84-185)	134 (120-171)	129 (116-154)	Normal – decreased: -0.43 (-22, 21), 0.999
Mean (95% CI)	137 (123, 150)	136 (122, 151)	137 (122, 152)	Increased – decreased: 0.02 (-22, 22), >0.999
Number of dogs	14	11	11	Increased – normal: 0.45 (-20, 21), 0.998
**DOCP dose (mg/kg)**				
Median (range)	1.15 (0.36-2.2)	0.76 (0.3-1.65)	0.58 (0.3-1.65)	Normal – decreased: -0.12 (-0.38, 0.14), 0.529
Mean (95% CI)	1.014 (0.829, 1.20)	0.884 (0.705, 1.06)	0.852 (0.671, 1.03)	Increased – decreased: -0.14 (-0.40, 0.13), 0.432
Number of dogs	16	17	19	Increased – normal: -0.02 (-0.19, 0.15), 0.952
**Prednisolone dose (mg/kg/d)**				
Median (range)	0.055 (0.03-0.1)	0.06 (0.03-0.09)	0.06 (0.03-0.1)	Normal – decreased: 0.01 (-0.01, 0.02), 0.577
Mean (95% CI)	0.057 (0.048, 0.066)	0.057 (0.048, 0.066)	0.057 (0.047, 0.066)	Increased – decreased: 0.01 (-0.01, 0.02), 0.629
Number of dogs	16	7	19	Increased – normal: 0.00 (-0.01, 0.01), 0.995
**Years since diagnosis**				
Median (range)	1.8 (0.42-6.1)	2.3 (0.42-9.7)	5.4 (0.6-9.4)	Normal – decreased: -0.06 (-1.1, 0.95), 0.989
Mean (95% CI)	3.33 (2.21, 4.45)	3.27 (2.17, 4.38)	3.24 (2.13, 4.35)	Increased – decreased: -0.09 (-1.1, 0.94), 0.975
Number of dogs	16	17	19	Increased – normal: -0.03 (-0.62, 0.56), 0.990
**Interval (d)**				
Median (range)	28 (21-36)	28 (28-56)	28 (28-56)	Normal – decreased: 1.5 (-3.2, 6.1), 0.731
Mean (95% CI)	28.5 (25.6, 31.4)	30.0 (27.2, 32.8)	30.0 (27.2, 32.9)	Increased – decreased: 1.5 (-3.2, 6.2), 0.720
Number of dogs	16	17	19	Increased – normal: 0.05 (-3.9, 4.0), >0.999

Abbreviations: *PRA* plasma renin activity, *Na/K ratio* sodium-to-potassium ratio, *USG* urine specific gravity, *DOCP* desoxycorticosterone pivalate, *CI* Confidence Interval, visits were stratified by PRA as decreased (below reference range), normal (within reference range), or increased (above reference range)

For each parameter, descriptive results are presented as median (range) with the corresponding number of visits per PRA category. In addition, estimated marginal means (EMMs) from the mixed-effects model are reported with 95% confidence intervals (CI). Between-group differences were evaluated using model-based contrasts (difference in EMMs) with 95% CIs

Statistical significance was defined as p < 0.05. Reference intervals were: potassium 4.3–5.3 mmol/L; sodium 145–152 mmol/L; Na/K ratio 28.6–33.7; creatinine 50–119 µmol/L. Significance codes: *p < 0.05; **p < 0.01; ***p < 0.001

USG data were also incomplete (*n* = 25), as owners frequently failed to provide samples and cystocentesis was not allowed for ethical reasons. Similarly, USG did not differ significantly among PRA groups (Table 2).

### Biochemical parameters, DOCP dose, prednisolone dose, injection interval and clinical impression

**Fig. 3:**
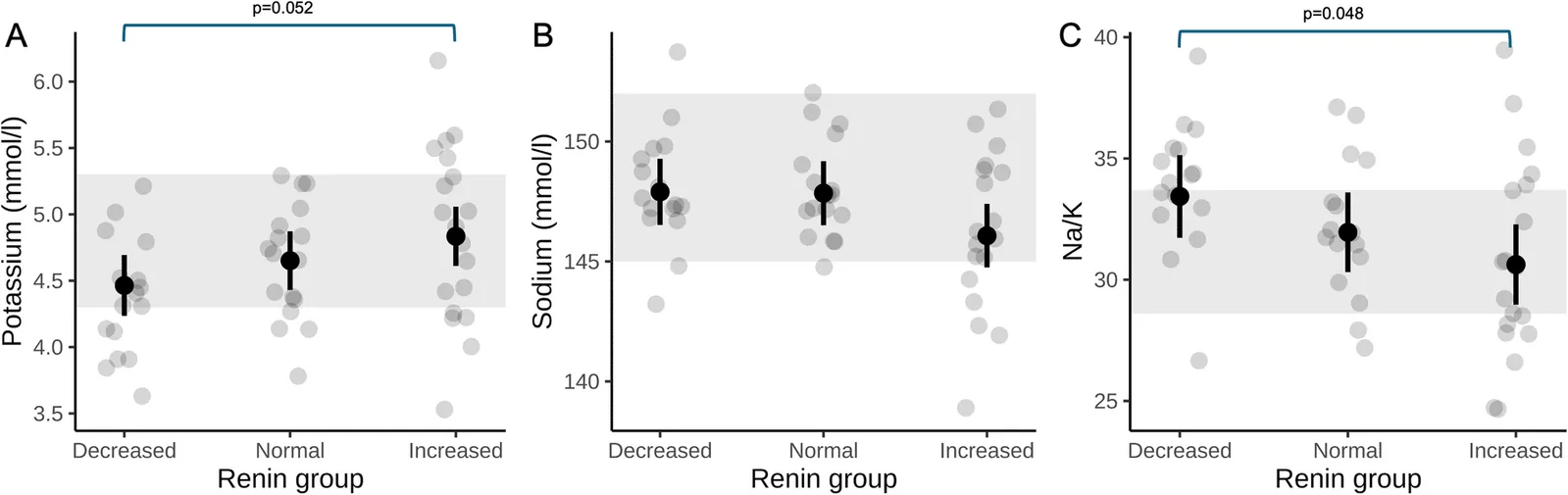
Scatterplots of potassium concentration (**A**), sodium concentration (**B**) and sodium-to-potassium (Na/K) ratio (**C**) in relationship with plasma renin activity (PRA) category (decreased: PRA below reference interval; normal: PRA within reference interval; increased: PRA above reference interval). Individual observations (visits) are shown as semi-transparent gray dots. Black circles represent estimated marginal means from linear mixed-effects models (accounting for repeated measurements within dogs) with vertical lines indicating 95% confidence intervals. The shaded gray area marks the normal reference interval for each electrolyte or the Na/K ratio. Statistical significance was defined as *p* < 0.05

**Fig. 4:**
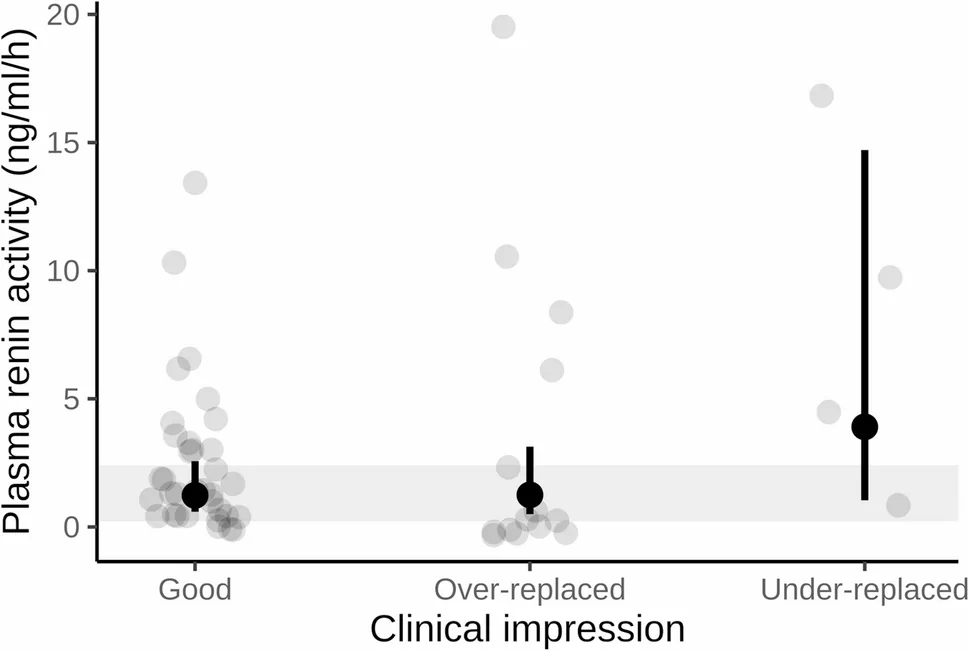
Scatterplots of the plasma renin activity (PRA) in dogs according to their clinical impression. The clinical impression was based on history, clinical signs, and electrolyte concentrations and was categorized into good (appropriate mineralocorticoid dose), under-replaced (suspected mineralocorticoid underdosing) or over-replaced (suspected mineralocorticoid overdosing). Individual observations (visits) are shown as semi-transparent gray dots. Black circles represent estimated marginal means from a Tweedie mixed-effects model (log link; accounting for repeated measurements within dog) with vertical lines indicating 95% confidence interval on the original PRA scale (back-transformed). The shaded gray area denotes the PRA reference interval

The biochemical and treatment parameters recorded during the 52 visits are summarized in Table 2. Comparison of biochemical and treatment parameters among the three PRA groups showed the following results: compared with visits with decreased PRA, visits with increased PRA showed a trend towards higher potassium concentrations (*p* = 0.052) and had a significantly lower sodium-to-potassium ratio (*p* = 0.048) (Table 2; Fig. 3). There was no significant difference in PRA among the three clinical impression categories (Table 3; Fig. 4).

**Table 3:** Association between plasma renin activity (PRA) and clinical impression in dogs on mineralocorticoid therapy

	Clinical impression			Comparison between groups
**Plasma renin activity (ng/ml/h)**	**Under-replaced**	**Good**	**Over-replaced**	**Contrast** ^1^ **(95% CI), ** *p* **-value**
Median (range)	7.02 (1.11–16.93)	1.66 (0-13.32)	0.2 (0.04–19.2)	Over-replaced – good; 1.02 (0.36, 2.88), > 0.999
Mean (95% CI)	3.91 (1.072, 14.24)	1.23 (0.601, 2.52)	1.25 (0.511, 3.07)	Under-replaced – good: 3.17 (0.81, 12.4), 0.117
Number of visits	4	34	14	Under-replaced – over-replaced: 3.12 (0.58, 16.9), 0.254

Visits were categorized by clinical impression as good (appropriate mineralocorticoid dose), under-replaced (suspected mineralocorticoid underdosing), or over-replaced (suspected mineralocorticoid overdosing), based on history, clinical signs, and electrolyte concentrations. For each group, PRA is presented descriptively as median (range) with the corresponding number of visits, and as estimated marginal means (EMMs) from a Tweedie mixed-effects model with a log link reported on the original PRA scale with 95% confidence intervals (CI) (back-transformed from the log scale)

^1^Pairwise comparisons between clinical impression categories are reported as model-based contrasts (ratio of EMMs) with 95% CIs and p-values (tests performed on the log scale). Statistical significance was defined as *p* < 0.05. Significance codes: *p < 0.05; **p < 0.01; ***p < 0.001

### Concurrent diseases

Documented concurrent conditions included chronic enteropathy (8 visits), leishmaniasis (1 visit), chronic kidney disease (8 visits), and splenic vein thrombosis of unknown etiology (4 visits). Corresponding adjunctive medications comprised budesonide (1 visit), allopurinol (1 visit), clopidogrel (4 visits), and sulfasalazine (2 visits).

**Fig. 5:**
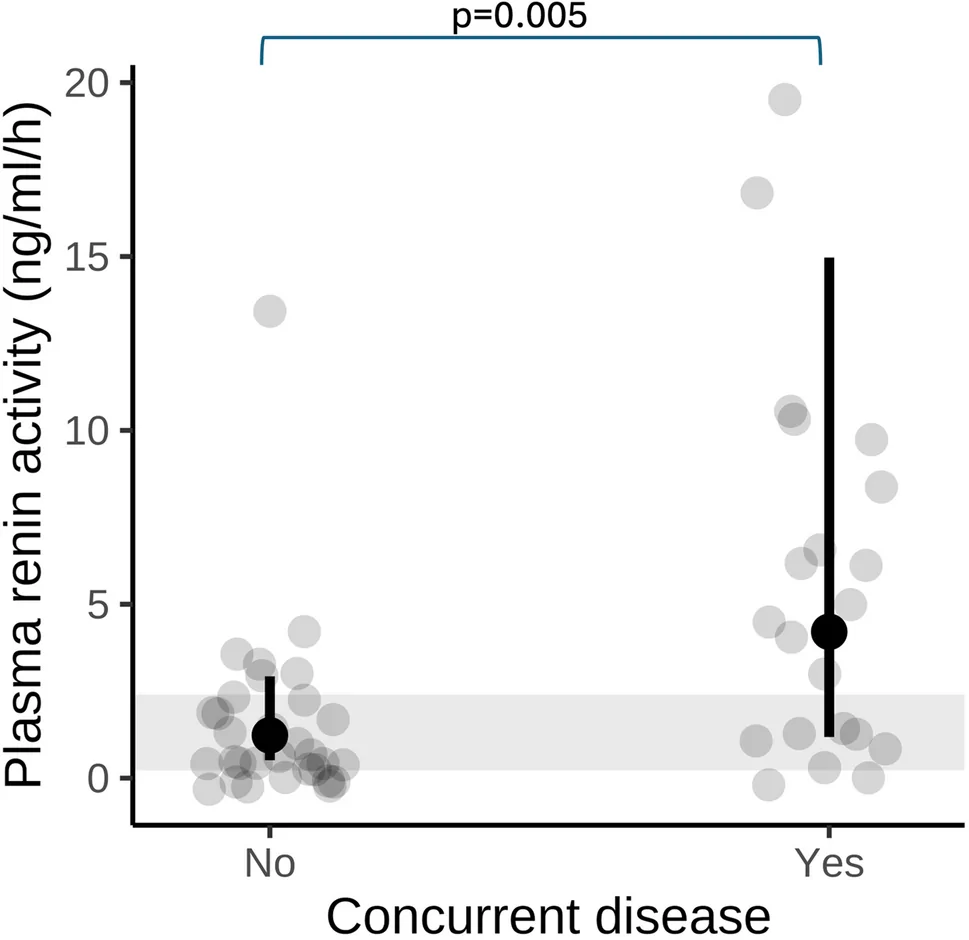
Scatterplots of the plasma renin activity (PRA) in dogs with and without concurrent disease. Individual observations (visits) are shown as semi-transparent gray dots. Black circles represent estimated marginal means from a Tweedie mixed-effects model (log link; accounting for repeated measurements within dog) with vertical lines indicating 95% confidence intervals on the original PRA scale (back-transformed). The shaded gray area marks the PRA reference interval. Statistical significance was defined as *p* < 0.05

PRA was significantly higher at visits during which concurrent disease was present (*p* = 0.005; Table 4; Fig. 5). Because chronic enteropathy and chronic kidney disease were the most frequently observed conditions, these were subsequently evaluated separately. For this purpose, visits were categorized according to the presence or absence of chronic enteropathy (present: *n* = 8; absent: *n* = 36) and, in a separate analysis, chronic kidney disease (present: *n* = 8; absent: *n* = 36). PRA did not differ significantly between visits with and without chronic enteropathy (*p* = 0.189) or chronic kidney disease (*p* = 0.077).

**Table 4:** Parameters significantly associated with concurrent disease

	**Concurrent disease** ^**+**^		**Comparison between groups**
**Plasma renin activity (ng/ml/h**)	**Yes**	**No**	**Contrast** ^1^ ** (95% CI), p-value**
Median (range) Mean (95% CI) Number of visits	4.23 (0-19.2) 4.21 (1.220, 14.52) 21	0.42 (0.04-13.32) 1.23 (0.527, 2.87) 31	Yes - No: 3.42** (1.44, 8.15), **0.005**
**Creatinine (** **μmol/L)**			
Median (range) Mean (95% CI) Number of visits	101 (64-167) 97 (85, 109) 19	78.5 (51-130) 83 (73, 93) 28	Yes - No: 14* (2.0, 27), **0.023**
**DOCP dose (mg/kg)**			
Median (range) Mean (95% CI) Number of visits	0.8 (0.32-1.65) 0.79 (0.59, 0.99) 21	1.0 (0.3-2.2) 0.995 (0.82, 1.2) 31	Yes - No: -0.2** (-0.34, -0.07), **0.004**

^**+**^Concurrent diseases included: chronic enteropathy; chronic kidney disease; SVT, splenic vein thrombosis; Leishmaniasis; Visits were grouped by concurrent disease status (Yes vs No). For each group, descriptive data are presented as median (range) with the corresponding number of visits. Model-based estimates are reported as estimated marginal means (EMMs with 95% confidence intervals (CIs): for plasma renin activity, from a Tweedie mixed-effects model with a log link and presented on the original scale (back-transformed); for creatinine and DOCP dose, from a robust linear mixed-effects model. ^1^Between-group differences (contrasts) were assessed using model-based ratios of EMMs (Yes/No), with 95% CIs and associated p-values (tests performed on the log scale) for Tweedie models and estimated mean difference for robust mixed-effects models. Statistical significance was defined as p < 0.05. Significance codes: *p < 0.05; **p < 0.01; ***p < 0.001

Besides the influence of concurrent diseases on PRA, the effects of concurrent diseases on other parameters were also evaluated. Creatinine was significantly higher (*p* = 0.023) and the DOCP dose significantly lower (*p* = 0.004) at visits during which a concurrent disease was present (Table 4). When chronic enteropathy and chronic kidney disease were evaluated separately, visits with chronic enteropathy were associated with a significantly higher potassium concentration (*p* = 0.003), a significantly lower Na/K ratio (*p* = 0.009), and significantly higher DOCP doses (p = < 0.001) (Table 5). Conversely, visits with chronic kidney disease were associated with significantly higher creatinine concentrations (p< 0.001) and significantly lower DOCP doses (p< 0.001) (Table 5).

**Table 5:** Parameters significantly associated with chronic enteropathy or chronic kidney disease

	**Chronic enteropathy**		**Comparison between groups**
**Potassium (mmol/L**)	**Yes**	**No**	**Contrast** ^1^ ** (95% CI), p-value**
Median (range) Mean (95% CI) Number of visits	5.1 (4.0-5.9) 5.03 (4.72, 5.34) 8	4.5 (3.8-5.4) 4.52 (4.35, 4.68) 36	Yes - No: 0.51** (0.18, 0.85), **0.003**
**Na/K ratio**			
Median (range) Mean (95% CI) Number of visits	27.8 (24.4-37) 29.4 (27, 31.8) 8	33 (27-38) 32.8 (31.6, 34.1) 36	Yes - No: -6.1** (-6.1, -0.85), **0.009**
**DOCP dose (mg/kg)**			
Median (range) Mean (95% CI) Number of visits	1.0 (0.32-1.65) 0.95 (0.79, 1.12) 8	0.85 (0.3-2.2) 0.92 (0.75, 1.08) 36	Yes - No: 0.04*** (0.02, 0.06), <**0.001**

	**Chronic kidney disease**		**Comparison between groups**
**Creatinine (** **μmol/L)**	**Yes**	**No**	**Contrast** ^1^ ** (95% CI), p-value**
Median (range) Mean (95% CI) Number of visits	123 (93-167) 123 (104, 142) 8^+^	81 (51-130) 83 (75, 92) 32	Yes - No: 40*** (21, 59), <**0.001**
**DOCP dose (mg/kg)**			
Median (range) Mean (95% CI) Number of visits	1.05 (0.43-1.53) 0.54 (0.33, 0.75) 8^+^	0.85 (0.3-2.2) 0.935 (0.75, 1.12) 36	Yes / No: -0.39*** (-0.5, -0.29), <**0.001**

Visits were grouped by concurrent disease status (Yes vs No). For each group, descriptive data are presented as median (range) with the corresponding number of visits. Model-based estimates are reported as estimated marginal means (EMMs) derived from a robust linear mixed-effects model or linear mixed-effects model (creatinine in chronic kidney disease), with 95% confidence intervals (CI). ^1^Between-group differences were assessed using model-based differences of EMMs (Yes-No), with 95% CIs and associated p-values. Statistical significance was defined as *p* < 0.05. Significance codes: *p < 0.05; **p < 0.01; ***p < 0.001. ^+^number of dogs = 2

### Visits with hyperkalemia and/or hyponatremia (possible under-replacement)

At 5 visits (4 dogs), serum potassium concentrations were above the upper reference limit. Hyponatremia was also present at two of these visits, whereas sodium concentrations were within the reference interval at the remaining three. Among the five visits with elevated potassium, three (60%) had elevated PRA, one (20%) had PRA within the reference interval, and one (20%) had decreased PRA. At the three visits with the highest potassium concentrations (≥ 5.6 mmol/L), PRA was consistently elevated (Supplementary Table 1).

A concurrent disease was documented or suspected at 4 of the 5 visits with increased potassium concentrations. Clinical signs observed at these visits included intermittent muscle stiffness (1 visit), lip smacking (1 visit), and intermittent vomiting and diarrhea (1 visit) (Supplementary Table 1).

At six visits (6 dogs), serum sodium concentrations were below the reference interval. At two of these visits, serum potassium was increased, whereas potassium concentrations were within normal limits at the remaining four. Elevated PRA was observed at five of the six visits (83.3%), while PRA was decreased at one visit (Supplementary Table 1).

A concurrent disease was documented or suspected at 5 of the 6 visits with decreased sodium concentration (the same visits in which PRA was elevated). Clinical signs documented during these visits included intermittent vomiting and diarrhea (2 visits), polyphagia with PU/PD and intermittent vomiting and diarrhea (1 visit), and lip smacking (1 visit) (Supplementary Table 1).

### Visits with hypokalemia and/or hypernatremia (possible over-replacement)

During 12 visits (11 dogs), serum potassium concentration was below the lower limit of the reference interval; at one visit, serum sodium concentration was also increased. PRA was decreased at six visits (50%), within the reference interval at four visits (33%), and elevated at two visits (17%).

A confirmed concurrent disease was documented at only one visit. Clinical signs observed during these visits included weight gain (1 visit), intermittent muscle stiffness (1 visit), and intermittent reduced appetite (1 visit) (Supplementary Table 2).

Despite consistent hypokalemia, PRA responses varied across visits. At the visit with the lowest potassium concentration (3.8 mmol/L), PRA was the highest (10.82 ng/mL/h). The dog associated with this visit did not show any clinical signs but presented to the emergency service the following day and was diagnosed with acute, severe pancreatitis. At that time, both potassium and sodium concentrations were within the reference interval.

At the single visit with concurrent hypernatremia and hypokalemia, PRA was markedly decreased. The dog associated with this visit was receiving a relatively high dose of DOCP (1.5 mg/kg) and did not show clinical signs; however, over-replacement was suspected (Supplementary Table 2).

### Divergence or agreement between the different parameters

In 25 (48%) visits, the clinical impression (based on history, clinical signs, and electrolyte concentrations) was consistent with the PRA measurement (e.g., the clinical impression suggested under-replacement, and PRA was increased). However, in 27 (52%) visits, the two assessments diverged. In 16 of these 27 visits (59%), PRA was increased (suggesting possible under-replacement), whereas the clinical impression was either considered adequate (*n* = 14) or indicated over-replacement (*n* = 4) (Supplementary Table 3). In 8 of 27 visits (30%), PRA was decreased (suggesting possible over-replacement), although the clinical impression was considered adequate. In the remaining 3 of 27 visits (11%), PRA was within the reference interval, while the clinical impression suggested over-replacement (*n* = 2) or under-replacement (*n* = 1) (Supplementary Table 3).

## Discussion

This study aimed to evaluate the clinical relevance of PRA as a marker of mineralocorticoid adequacy in dogs with hypoadrenocorticism treated with DOCP and glucocorticoids. Previous studies show that PRA is elevated in untreated disease and suppressed with mineralocorticoid treatment, with DOCP causing stronger suppression than fludrocortisone [4, 5]. A randomized trial found frequent PRA over-suppression at the standard DOCP dose, whereas it was less common with a low-dose protocol, suggesting PRA may help detect subclinical overdosing [18]. A recent pharmacokinetic study of DOCP extended these observations by showing that increases in PRA often preceded electrolyte abnormalities by one to two weeks in most dogs, and that serum electrolyte concentrations overestimated the duration of DOCP action by a mean of 10 days compared with PRA [19]. Further evidence comes from an earlier study in which DOCP dosing intervals were individualized [20]. In that study, serum electrolyte concentrations remained within the reference interval for more than 70 days, and in some dogs for more than 100 days, despite pharmacokinetic data from a recent study indicating that serum DOC concentrations, the active metabolite of DOCP, typically fall below the limit of quantification much earlier. This suggests that many of these dogs may have been mineralocorticoid-deficient toward the end of their dosing intervals despite unremarkable electrolyte values [20]. Collectively, these findings support the use of PRA as an adjunct to electrolyte measurements and clinical assessment when titrating mineralocorticoid treatment.

In our study, PRA correlated moderately positively with potassium and moderately negatively with sodium and the sodium-to-potassium ratio, but the overall clinical assessment (based on history, physical examination, and electrolyte findings) diverged in more than half of the visits. This discordance likely reflects the complexity of assessing mineralocorticoid status and the fact that PRA and electrolyte-based clinical assessment may not always align. Cases of elevated PRA despite unremarkable electrolyte concentrations and an unremarkable clinical assessment may reflect underlying subclinical mineralocorticoid deficiency. Conversely, subclinical overtreatment may also not reliably be captured by electrolyte measurements. In the above-mentioned randomized study, Na/K ratios were frequently within the reference interval or at its upper end in dogs treated with standard-dose DOCP, while PRA was simultaneously suppressed in the majority of these assessments [18]. A comparable pattern was observed in an earlier study comparing DOCP with fludrocortisone, in which PRA was often completely suppressed in DOCP-treated dogs despite electrolyte concentrations within the reference interval [5]. Discordance between PRA and electrolytes in the present study should therefore not be interpreted as a limitation of PRA alone, but rather as a reflection of the complementary, and at times divergent, information that these two parameters can provide.

A confounding factor influencing PRA independent of the DOCP dose is the presence of concurrent disease. PRA was significantly higher at visits during which concurrent disease was present. Concurrent disease may also alter electrolyte concentrations independently of mineralocorticoid replacement, as observed in this study, and any additional treatments may further influence both parameters. However, due to the small number of cases with concurrent diseases and additional treatment, this effect could not be fully evaluated. Therefore, in dogs with concurrent disease, both electrolyte concentrations and PRA concentrations should be interpreted carefully when assessing treatment adequacy.

Another factor that may also influence PRA and electrolyte concentrations by affecting water intake and hydration status is concomitant glucocorticoid therapy and its dosing. In this study, however, no significant correlation was found between prednisolone dose and PRA, nor was there an association with PRA categories, concurrent disease, or any subgroup thereof. It is also noteworthy that prednisolone doses were very similar across PRA categories, reflecting our institutional practice of titrating to the lowest effective dose. Therefore, we assume that prednisolone dosing did not substantially influence the evaluated parameters in this cohort, although its potential impact should be considered in animals receiving higher glucocorticoid supplementation.

At a considerable number of visits, electrolyte concentrations were within the reference interval and the dogs seemed clinically stable, yet PRA was elevated. Several factors may explain this finding. Subclinical under-replacement, for example, could lead to early activation of the RAAS before overt electrolyte changes become apparent. This hypothesis is consistent with the pharmacokinetic findings discussed above [19]. Based on the available data, it remains unclear whether an increase in mineralocorticoid dose is warranted in cases with elevated PRA despite normal electrolytes and unremarkable clinical assessment. Although in human medicine, PRA is considered a more sensitive marker of under-replacement [21], attempts to normalize PRA in otherwise clinically stable patients may risk overtreatment [22]. At the same time, relying on electrolyte concentrations alone carries the opposite risk of missing subclinical under-replacement, as discussed above. Balancing these two possibilities in the individual dog remains challenging and illustrates that neither parameter alone may consistently be sufficient. Although direct comparison with human medicine is limited because treatment regimens differ substantially, the available human literature likewise suggests that no single marker fully reflects mineralocorticoid replacement adequacy. Human studies highlight that renin, electrolytes, blood pressure, and clinical findings may each provide complementary information [7, 21, 23]. This supports a cautious interpretation of discordant findings and argues against relying on either PRA or electrolyte concentrations alone.

Importantly, elevated PRA in the presence of normal electrolyte concentrations may also have alternative explanations. Beyond the effect of concurrent disease discussed above, factors related to the sampling context and physiological state, such as timing within the DOCP dosing interval, recent dose adjustments, hydration status, sodium intake, stress and feeding may further contribute to variability in PRA measurements [24, 25]. While pre-analytical handling can influence renin measurements, this is unlikely to account for the variability observed in the present study, as samples were processed and stored under standardized conditions designed to preserve renin stability.

Our results revealed no significant association or correlation between PRA and USG or blood pressure measurements. A significant negative correlation was found only between USG and the DOCP dose. An association between urine specific gravity and DOCP dosage has also been shown in an earlier study [18]. In that study, dogs receiving a higher DOCP dose had significantly lower USG values compared with those receiving a lower dose [18]. The fact that this difference is not reflected in a corresponding change in PRA in our study could have several explanations. USG was not measured at every visit, which limits statistical power. Variability in individual patient responses to DOCP treatment, including differences in renal sensitivity, may also contribute to the lack of a consistent relationship between PRA and USG. Furthermore, other factors influencing urine concentration or PRA, such as hydration status, concurrent medications, or underlying comorbidities, could have confounded the associations in this study. Including blood pressure measurement at every re-check was very challenging, mainly because these were routine visits for stable and highly active dogs within a tightly scheduled clinical setting. Successful measurements were often not possible, which certainly limited the statistical power for this parameter.

Several limitations should be considered when interpreting the findings of the present study. The sample size was relatively small, especially within the electrolyte- and PRA-defined subgroups, which reduced statistical power and precision. Differences in treatment stage, treatment duration, and individualized DOCP regimens likely contributed to variability between visits. The presence of concurrent disease may have further increased heterogeneity between cases, as these conditions can independently affect both electrolytes and RAAS activity. Sampling was performed only on the day of scheduled DOCP administration and therefore captured only a limited aspect of RAAS dynamics. Clinical assessments were not standardized and were based on the clinician’s recorded impressions. This assessment relied heavily on electrolyte concentrations, which are available immediately, whereas PRA results were not. This may have introduced observer bias. Consistent with the pre-analytical and physiological factors discussed above, time of day and feeding status were not standardized in the present study, which may have contributed to variability in PRA measurements. In addition, other components of the RAAS, such as aldosterone and angiotensin I and II, were not measured, which limits mechanistic interpretation.

## Conclusions

This study highlights the complexity of PRA as a monitoring tool in dogs with hypoadrenocorticism treated with DOCP. PRA showed moderate correlations with electrolyte concentrations and DOCP dose, but also considerable variability, with discordance between PRA and electrolyte-based clinical assessment in more than half of the visits. Because neither parameter can currently be regarded as a definitive reference standard for mineralocorticoid adequacy, these findings should be viewed as reflecting that PRA and electrolytes provide complementary and sometimes different information, rather than indicating a weakness of either measure alone. PRA, potassium concentrations, and the Na/K ratio were additionally affected by concurrent disease, indicating that comorbidities may influence both PRA and electrolyte status independently of mineralocorticoid treatment. Overall, PRA may provide additional information for monitoring mineralocorticoid adequacy but should be interpreted together with electrolytes and the overall clinical picture. Future prospective longitudinal studies with repeated and standardized measurements of PRA and electrolytes are needed to clarify the clinical value of PRA-guided treatment adjustments in dogs with hypoadrenocorticism.

## Supplementary Information

Supplementary Material 1. Supplementary Table 1: Individual clinical variables, DOCP and prednisolone doses of dogs with hyperkalemia and/or hyponatremia

Supplementary Material 2. Supplementary Table 2: Individual clinical variables, DOCP and prednisolone doses of dogs with hypokalemia and/or hypernatremia

Supplementary Material 3. Supplementary Table 3: Divergence or agreement of the clinical impression and plasma renin activity.

## Data Availability

The datasets used and/or analysed during the current study are available form the corresponding authors on reasonable request.

## Abbreviations

PRA: plasma renin activity
DOCP: desoxycorticosterone pivalate
ELISA: enzyme-linked immunosorbent assay
RAAS: renin-angiotensin-aldosterone system
USG: urine specific gravity
