# Supplementary material for: Evaluating plasma renin activity as a monitoring parameter for mineralocorticoid treatment in dogs with primary hypoadrenocorticism
Source: BMC Vet Res. 2026 Aug 8;22:668. doi: 10.1186/s12917-026-05645-y (PMC13737664; doi:10.1186/s12917-026-05645-y)
Supplement: Supplementary file 1 [file 12917_2026_5645_MOESM1_ESM.docx]

**Supplementary Table 1**

| **Dog** | **Potassium (mmol/L)** | **Sodium (mmol/L)** | **Na/K ratio** | **Creatinine (umol/l)** | **PRA (ng/mL/h)** | **DOCP dose (mg/kg)** | **Injection interval (d)** | **Prednisolone dose (mg/kg/d)** | **Years since dx** | **Clinical signs** | **Clinical impression** | **Concurrent diseases** |
| --- | --- | --- | --- | --- | --- | --- | --- | --- | --- | --- | --- | --- |
| 3 | 5.1 | **142** ↓ | **28**↓ | 78 | **5.85** ↑ | 0.5 | 33 | 0.1 | 6.4 | Intermittent vomiting and diarrhea | Good | CE |
| 6 | **5.6** ↑ | 149 | **27**↓ | **142**↑ | **3.00** ↑ | 1.65 | 28 | 0.08 | 0.6 | No | Good | CE |
|  | **5.5** ↑ | 151 | **27**↓ | **120**↑ | 1.11 | 1.65 | 28 | 0.06 | 0.8 | Intermittent muscle stiffness | Under-replaced | CE |
| 11 | 5.1 | **142** ↓ | **28**↓ | nd | **9.81** ↑ | 1.1 | 28 | 0.05 | 8.4 | Polyphagia, PU/PD, Intermittent vomiting and diarrhea | Under-replaced | CE |
| 14 | **5.6** ↑ | **139** ↓ | **25**↓ | **131**↑ | **16.93** ↑ | 1.3 | 28 | 0.08 | 8.7 | Lip-smacking | Under-replaced | CKD |
| 17 | **5.9** ↑ | **144** ↓ | **24**↓ | 71 | **4.23** ↑ | 1.07 | 28 | 0.07 | 3.5 | Intermittent vomiting and diarrhea | Under-replaced | CE susp. |
| 19 | 4.7 | **143** ↓ | 30 | 101 | **19.2** ↑ | 0.37 | 30 | 0.05 | 5.5 | No | Over-replaced | Splenic vein thrombosis |
| 24 | **5.4** ↑ | 145 | **27**↓ | 64 | **0.08** ↓ | 1.65 | 28 | 0.05 | 1.2 | No | Good |  |
| 25 | 4.3 | **143** ↓ | 33 | 76 | **0.21** ↓ | 0.7 | 28 | 0.1 | 3.1 | No | Good |  |

Abbreviations: Na/K ratio, sodium-to-potassium ratio; PRA, plasma renin activity; USG, urine specific gravity; DOCP, desoxycorticosterone pivalate; dx, diagnosis; nd, not determined; PU/PD, polyuria and polydipsia; CE; chronic enteropathy; CKD, chronic kidney disease; susp., suspected; Reference ranges: PRA: 0.22–2.4 ng/mL/h; potassium: 4.3–5.3 mmol/L; sodium: 145–152 mmol/L; Na/K ratio: 28.6-33.7; creatinine: 50-119 µmol/L

Color coding: red color: potassium above reference range, sodium below reference range; ↓: value below the reference range; ↑: value above the reference range.
