# Supplementary material for: Evaluating plasma renin activity as a monitoring parameter for mineralocorticoid treatment in dogs with primary hypoadrenocorticism
Source: BMC Vet Res. 2026 Aug 8;22:668. doi: 10.1186/s12917-026-05645-y (PMC13737664; doi:10.1186/s12917-026-05645-y)
Supplement: Supplementary file 2 [file 12917_2026_5645_MOESM2_ESM.docx]

**Supplementary Table 2**

| **Dog** | **Potassium (mmol/L)** | **Sodium (mmol/L)** | **Na/K ratio** | **Creatinine (umol/l)** | **PRA (ng/mL/h)** | **DOCP dose (mg/kg)** | **Injection interval (d)** | **Prednisolone dose (mg/kg/d)** | **Years since dx** | **Clinical signs** | **Clinical impression** | **Concurrent diseases** |
| --- | --- | --- | --- | --- | --- | --- | --- | --- | --- | --- | --- | --- |
| 5 | **4.1 ↓** | 152 | **37**↑ | nd | 2.29 | 0.68 | 28 | 0.06 | 0.7 | Weight gain | Over-replaced |  |
| 13 | **4.2 ↓** | 148 | 35 | 93 | **0.19 ↓** | 1.0 | 28 | 0.05 | 0.4 | Intermittent muscle stiffness | Over-replaced | CE |
| 15  15 | **4.0 ↓** | 148 | **37**↑ | 61 | **2.69 ↑** | 0.3 | 30 | 0.09 | 1.5 | Intermittent reduced appetite | Good |  |
|  | **4.0 ↓** | 148 | **37**↑ | 67 | 1.48 | 0.3 | 56 | 0.07 | 2.3 | No | Good | CE |
| 16 | **4.2 ↓** | 150 | **36**↑ | 102 | **0.04 ↓** | 1.2 | 28 | 0.06 | 1.1 | No | Over-replaced |  |
| 19 | **3.8 ↓** | 151 | **40**↑ | 108 | **10.82 ↑** | 0.37 | 30 | 0.05 | 5.4 | No | Over-replaced | Splenic vein thrombosis |
| 20 | **4.1 ↓** | 147 | **36**↑ | 54 | **0.07 ↓** | 0.96 | 28 | 0.06 | 4.9 | No | Good |  |
| 22 | **4.2 ↓** | 147 | **35**↑ | 51 | 1.75 | 1.48 | 28 | 0.07 | 4.7 | No | Good |  |
| 23 | **4.2 ↓** | 147 | **35**↑ | 76 | 0.45 | 1.2 | 28 | 0.09 | 0.9 | No | Over-replaced |  |
| 26 | **3.9 ↓** | **154 ↑** | **39**↑ | 75 | **0.04 ↓** | 1.5 | 28 | 0.1 | 1.8 | No | Over-replaced |  |
| 26 | **4.2 ↓** | 148 | **35**↑ | 74 | **0.04 ↓** | 1.1 | 28 | 0.08 | 2.8 | No | Over-replaced |  |
| 28 | **4.1 ↓** | 150 | **37**↑ | 86 | **0.21 ↓** | 0.6 | 28 | 0.04 | 1.0 | No | Over-replaced |  |

Abbreviations: Na/K ratio, sodium-to-potassium ratio; PRA, plasma renin activity; USG, urine specific gravity; DOCP, desoxycorticosterone pivalate; dx, diagnosis; PU/PD, polyuria and polydipsia; CE; chronic enteropathy; CKD, chronic kidney disease; susp., suspected; Reference ranges: PRA: 0.22–2.4 ng/mL/h; potassium: 4.3–5.3 mmol/L; sodium: 145–152 mmol/L; Na/K ratio: 28.6-33.7; creatinine: 50-119 µmol/L.

Color coding: green color: potassium below reference range, sodium above reference range. ↓: value below the reference range; ↑: value above the reference range.
