# Supplementary material for: Evaluating plasma renin activity as a monitoring parameter for mineralocorticoid treatment in dogs with primary hypoadrenocorticism
Source: BMC Vet Res. 2026 Aug 8;22:668. doi: 10.1186/s12917-026-05645-y (PMC13737664; doi:10.1186/s12917-026-05645-y)
Supplement: Supplementary file 3 [file 12917_2026_5645_MOESM3_ESM.docx]

**Supplementary Table 3**

|  | **PRA**  **(ng/mL/h)** | **Potassium**  **(mmol/L)** | **Sodium**  **(mmol/L)** | **Na/K ratio** | **Creatinine**  **(µmol/L)** | **DOCP dose**  **(mg/kg)** | **Injection interval (d)** | **Prednisolone**  **dose**  **(mg/kg/d)** | **Years since dx** | **Clinical signs** | **Clinical**  **Impression*** | **Concurrent disease** |
| --- | --- | --- | --- | --- | --- | --- | --- | --- | --- | --- | --- | --- |
| **Dog 1** |  |  |  |  |  |  |  |  |  |  |  |  |
| Visit 1 | 0.21** | 4.8 | 147 | 31 | 81 | 1.32 | 28 | 0.04 | 2.8 | PU/PD | Good |  |
| **Dog 2** |  |  |  |  |  |  |  |  |  |  |  |  |
| Visit 1 | 13.32** | 4.8 | 146 | 30 | 89 | 0.9 | 35 | 0.06 | 6.8 |  | Good |  |
| **Dog 3** |  |  |  |  |  |  |  |  |  |  |  |  |
| Visit 1 | 5.85** | 5.1 | 142 | 28 | 78 | 0.5 | 33 | 0.1 | 6.4 | Intermittent vomiting and diarrhea | Good | CE |
| Visit 2 | 3.84** | 5.0 | 145 | 29 | 64 | 0.5 | 33 | 0.1 | 6.9 | Intermittent vomiting and diarrhea | Good | CE |
| **Dog 4** |  |  |  |  |  |  |  |  |  |  |  |  |
| Visit 1 | 3.06** | 4.5 | 146 | 32 | 86 | 0.58 | 28 | 0.06 | 1.1 | Intermittent reduced appetite | Good |  |
| Visit 2 | 1.04 | 4.7 | 148 | 31 | 88 | 0.58 | 28 | 0.06 | 1.4 | PU/PD | Good |  |
| **Dog 5** |  |  |  |  |  |  |  |  |  |  |  |  |
| Visit 1 | 2.29** | 4.1 | 152 | 37 | nd | 0.68 | 28 | 0.06 | 0.7 | Weight gain | Over-repl. |  |
| Visit 2 | 5.77** | 4.3 | 151 | 35 | 167 | 0.43 | 28 | 0.06 | 0.9 | Weight gain | Over-repl. | CKD |
| **Dog 6** |  |  |  |  |  |  |  |  |  |  |  |  |
| Visit 1 | 3.00** | 5.6 | 149 | 27 | 142 | 1.65 | 28 | 0.08 | 0.6 |  | Good | CE |
| Visit 2 | 1.11** | 5.5 | 151 | 27 | 120 | 1.65 | 28 | 0.06 | 0.8 | Intermittent muscle stiffness | Under-repl. | CE |
| **Dog 7** |  |  |  |  |  |  |  |  |  |  |  |  |
| Visit 1 | 1.84 | 4.4 | 146 | 33 | 69 | 0.76 | 28 | 0.06 | 0.7 |  | Good |  |
| **Dog 8** |  |  |  |  |  |  |  |  |  |  |  |  |
| Visit 1 | 0.14** | 4.6 | 149 | 32 | 114 | 1.25 | 36 | 0.06 | 0.8 | Intermittent vomiting and diarrhea | Good |  |
| **Dog 9** |  |  |  |  |  |  |  |  |  |  |  |  |
| Visit 1 | 0.07 | 4.4 | 149 | 34 | 75 | 0.8 | 28 | 0.08 | 1.8 |  | Over-repl. | Leishmaniasis |
| **Dog 10** |  |  |  |  |  |  |  |  |  |  |  |  |
| Visit 1 | 0.09** | 4.5 | 147 | 33 | nd | 2.2 | 21 | 0.05 | 0.42 |  | Good |  |
| Visit 2 | 0.04 | 4.3 | 147 | 34 | 88 | 1.6 | 28 | 0.04 | 0.9 | Polyphagia | Over-repl. |  |
| **Dog 11** |  |  |  |  |  |  |  |  |  |  |  |  |
| Visit 1 | 9.81 | 5.1 | 142 | 28 | nd | 1.1 | 28 | 0.05 | 8.4 | Polyphagia, PU/PD, Intermittent vomiting and diarrhea | Under-repl. | CE |
| **Dog 12** |  |  |  |  |  |  |  |  |  |  |  |  |
| Visit 1 | 0.26 | 4.7 | 147 | 31 | nd | 1.0 | 28 | 0.05 | 4.5 |  | Good |  |
| **Dog 13** |  |  |  |  |  |  |  |  |  |  |  |  |
| Visit 1 | 0.19 | 4.2 | 148 | 35 | 93 | 1.0 | 28 | 0.05 | 0.4 | Intermittent muscle stiffness | Over-repl. | CE |
| **Dog 14** |  |  |  |  |  |  |  |  |  |  |  |  |
| Visit 1 | 5.31** | 5.1 | 147 | 29 | 93 | 1.3 | 28 | 0.08 | 8.4 | PU/PD | Good | CKD susp. |
| Visit 2 | 6.38** | 5.1 | 145 | 28 | 98 | 1.3 | 28 | 0.08 | 8.7 |  | Good | CKD |
| Visit 3 | 16.93 | 5.6 | 139 | 25 | 131 | 1.3 | 28 | 0.08 | 9.4 | Lip-smacking | Under-repl. | CKD |
| Visit 4 | 0.99 | 5.0 | 148 | 30 | 111 | 1.5 | 28 | 0.08 | 9.7 |  | Good | CKD |
| **Dog 15** |  |  |  |  |  |  |  |  |  |  |  |  |
| Visit 1 | 2.69** | 4.0 | 148 | 37 | 61 | 0.3 | 30 | 0.09 | 1.5 | Intermittent reduced appetite | Good |  |
| Visit 2 | 3.46** | 4.4 | 149 | 34 | 84 | 0.3 | 56 | 0.09 | 1.9 | Polyphagia | Good |  |
| Visit 3 | 1.48 | 4.0 | 148 | 37 | 67 | 0.3 | 56 | 0.07 | 2.3 |  | Good | CE |
| **Dog 16** |  |  |  |  |  |  |  |  |  |  |  |  |
| Visit 1 | 4.39** | 4.4 | 150 | 34 | 109 | 1.2 | 28 | 0.05 | 0.7 |  | Good |  |
| Visit 2 | 0.04 | 4.2 | 150 | 36 | 102 | 1.2 | 28 | 0.06 | 1.1 |  | Over-repl. |  |
| Visit 3 | 8.67** | 4.3 | 149 | 35 | 153 | 0.8 | 28 | 0.06 | 1.6 |  | Over-repl. | CKD susp. |
| Visit 4 | 0.00** | 4.4 | 151 | 34 | 115 | 0.7 | 28 | 0.06 | 1.8 |  | Good | CKD |
| Visit 5 | 1.67 | 4.6 | 148 | 32 | 130 | 0.7 | 28 | 0.06 | 2.2 |  | Good | CKD |
| **Dog 17** |  |  |  |  |  |  |  |  |  |  |  |  |
| Visit 1 | 1.78 | 4.6 | 147 | 32 | 70 | 1.0 | 28 | 0.07 | 2.3 | Soft stool | Good |  |
| Visit 2 | 4.23 | 5.9 | 144 | 24 | 71 | 1.07 | 28 | 0.07 | 3.5 | Intermittent vomiting and diarrhea | Under-repl. | CE susp. |
| **Dog 18** |  |  |  |  |  |  |  |  |  |  |  |  |
| Visit 1 | 0.42 | 5.0 | 146 | 29 | 98 | 0.51 | 28 | 0.03 | 3.4 |  | Good |  |
| Visit 2 | 3.31** | 5.3 | 146 | 28 | 96 | 0.51 | 28 | 0.03 | 3.9 |  | Good |  |
| Visit 3 | 1.09 | 4.6 | 151 | 33 | 67 | 0.51 | 28 | 0.03 | 4.9 |  | Good |  |
| **Dog 19** |  |  |  |  |  |  |  |  |  |  |  |  |
| Visit 1 | 1.03 | 4.7 | 149 | 32 | 100 | 0.38 | 28 | 0.05 | 4.6 |  | Good | SVT |
| Visit 2 | 10.82** | 3.8 | 151 | 40 | 108 | 0.37 | 30 | 0.05 | 5.4 |  | Over-repl. | SVT |
| Visit 3 | 19.2** | 4.7 | 143 | 30 | 101 | 0.37 | 30 | 0.05 | 5.5 |  | Over-repl. | SVT |
| Visit 4 | 10.6** | 4.7 | 146 | 31 | nd | 0.37 | 30 | 0.05 | 5.7 | Lip-smacking | Good | SVT |
| **Dog 20** |  |  |  |  |  |  |  |  |  |  |  |  |
| Visit 1 | 0.07** | 4.1 | 147 | 36 | 54 | 0.96 | 28 | 0.06 | 4.9 |  | Good |  |
| **Dog 21** |  |  |  |  |  |  |  |  |  |  |  |  |
| Visit 1 | 0.21** | 4.6 | 147 | 32 | 130 | 0.36 | 28 | 0.03 | 5.3 |  | Good |  |
| **Dog 22** |  |  |  |  |  |  |  |  |  |  |  |  |
| Visit 1 | 1.75 | 4.2 | 147 | 35 | 51 | 1.48 | 28 | 0.07 | 4.7 |  | Good |  |
| Visit 2 | 1.64 | 4.5 | 145 | 32 | 57 | 1.23 | 28 | 0.05 | 5.3 |  | Good |  |
| Visit 3 | 0.07 | 4.3 | 147 | 34 | 67 | 1.43 | 28 | 0.04 | 6.1 | Danndruff | Over-repl. |  |
| **Dog 23** |  |  |  |  |  |  |  |  |  |  |  |  |
| Visit 1 | 0.34 | 4.9 | 150 | 31 | 70 | 1.2 | 28 | 0.09 | 0.42 |  | Good |  |
| Visit 2 | 0.45** | 4.2 | 147 | 35 | 76 | 1.2 | 28 | 0.09 | 0.9 |  | Over-repl. |  |
| **Dog 24** |  |  |  |  |  |  |  |  |  |  |  |  |
| Visit 1 | 0.08** | 5.4 | 145 | 27 | 64 | 1.65 | 28 | 0.05 | 1.2 |  | Good |  |
| **Dog 25** |  |  |  |  |  |  |  |  |  |  |  |  |
| Visit 1 | 0.21** | 4.3 | 143 | 33 | 76 | 0.7 | 28 | 0.1 | 3.1 |  | Good |  |
| **Dog 26** |  |  |  |  |  |  |  |  |  |  |  |  |
| Visit 1 | 0.04 | 3.9 | 154 | 39 | 75 | 1.5 | 28 | 0.1 | 1.8 |  | Over-repl. |  |
| Visit 2 | 0.04 | 4.2 | 148 | 35 | 74 | 1.1 | 28 | 0.08 | 2.8 |  | Over-repl. |  |
| **Dog 27** |  |  |  |  |  |  |  |  |  |  |  |  |
| Visit 1 | 2.40 | 5.2 | 146 | 28 | 81 | 0.63 | 28 | 0.08 | 7.4 |  | Good |  |
| **Dog 28** |  |  |  |  |  |  |  |  |  |  |  |  |
| Visit 1 | 0.21 | 4.1 | 150 | 37 | 86 | 0.6 | 28 | 0.04 | 1.0 |  | Over-repl. |  |

Abbreviations: PRA, plasma renin activity; Na/K ratio, sodium-to-potassium ratio; DOCP, desoxycorticosterone pivalate; nd, not determined; PU/PD, polyuria and polydipsia; over-repl., over-replaced - suspected mineralocorticoid overdosing leading to dose decrease of DOCP; Under-repl., under-replaced - suspected mineralocorticoid underdosing leading to dose increase of DOCP; CE; chronic enteropathy; CKD, chronic kidney disease; susp., suspected; SVT, splenic vein thrombosis;

Reference ranges: PRA: 0.22–2.4 ng/mL/h; potassium: 4.3–5.3 mmol/L; sodium: 145–152 mmol/L; Na/K ratio: 28.6-33.7; creatinine: 50-119 µmol/L.

Color coding: red color: renin activity or potassium above reference range, sodium below reference range; green color: renin activity or potassium below reference range, sodium above reference range.

* clinical impression: combination of history, clinical sings and electrolyte concentrations
** divergence between clinical impression and renin activity level
